# Metabolomic analysis of aqueous humor reveals potential metabolite biomarkers for differential detection of macular edema

**DOI:** 10.1186/s40662-023-00331-8

**Published:** 2023-04-01

**Authors:** Dan Jiang, Congcong Yan, Lina Ge, Chun Yang, Ying Huang, Yau Kei Chan, Chonghua Chen, Wei Chen, Meng Zhou, Bing Lin

**Affiliations:** 1 National Clinical Research Center for Ocular Diseases, Eye Hospital, Wenzhou Medical University, Wenzhou, 325027 China; 2 Department of Ophthalmology, LKS Faculty of Medicine, The University of Hong Kong, Hong Kong, SAR China

**Keywords:** Macular edema, Liquid chromatography-mass spectrometry, Metabolic biomarkers, Metabolomics

## Abstract

**Background:**

Macular edema (ME) is a major complication of retinal disease with multiple mechanisms involved in its development. This study aimed to investigate the metabolite profile of aqueous humor (AH) in patients with ME of different etiologies and identify potential metabolite biomarkers for early diagnosis of ME.

**Methods:**

Samples of AH were collected from 60 patients with ME and 20 age- and sex-matched controls and analyzed by liquid chromatography-mass spectrometry (LC/MS)-based metabolomics. A series of univariate and multivariate statistical analyses were performed to identify differential metabolites and enriched metabolite pathways.

**Results:**

The metabolic profile of AH differed significantly between ME patients and healthy controls, and differentially expressed metabolites were identified. Pathway analysis revealed that these differentially expressed metabolites are mainly involved in lipid metabolism and amino acid metabolism. Moreover, significant differences were identified in the metabolic composition of AH from patients with ME due to different retinal diseases including age-related macular degeneration (AMD-ME), diabetic retinopathy (DME) and branch retinal vein occlusion (BRVO-ME). In total, 39 and 79 etiology-specific altered metabolites were identified for AMD-ME and DME, respectively. Finally, an AH-derived machine learning-based diagnostic model was developed and successfully validated in the test cohort with an area under the receiver operating characteristic (ROC) curve of 0.79 for AMD-ME, 0.94 for DME and 0.77 for BRVO-ME.

**Conclusions:**

Our study illustrates the potential underlying metabolic basis of AH of different etiologies across ME populations. We also identify AH-derived metabolite biomarkers that may improve the differential diagnosis and treatment stratification of ME patients with different etiologies.

**Supplementary Information:**

The online version contains supplementary material available at 10.1186/s40662-023-00331-8.

## Background

Macular edema (ME) is the final common pathway of many intraocular and systemic injuries and diseases such as intraocular surgery, veno-occlusive disease, diabetic retinopathy (DR), and wet age-related macular degeneration (AMD). The diagnosis of retinal disease depends largely on digital imaging such as optical coherence tomography (OCT) and fluorescein angiography (FA). Nonetheless, most OCT systems have a limited imaging range with the macula and optic disc being the main imaging positions. However, peripheral changes may be missed, especially in the early stage of disease [1, 2]. Current sophisticated imaging techniques reveal only structural changes to the macula and cannot provide information about the pathophysiology at a cellular level [3, 4]. Furthermore, the pathophysiological characteristics of ME have not been completely defined. The development of precision medicine, including the use of quantitative indices on omics methods, provides a diagnosis/treatment of ME.

ME is caused by various retinal diseases so study of the molecular mechanisms of ME and identification of precise therapeutic targets for ME is important [4, 5]. The occurrence and progression of ME have been associated with the vascular endothelial growth factor (VEGF) family and inflammatory factors [6, 7]. Unfortunately, there is clinical evidence that intravitreal anti-VEGF therapy is ineffective in some cases of diabetic macular edema (DME) [8]. Further research into the exact intraocular molecular changes in ME of different etiologies and development of new therapeutic targets are needed to treat cases of ME that do not respond to anti-VEGF therapy. Metabolomics is an emerging discipline and defined as the comprehensive assessment of low-molecular-weight (< 1 kDa) endogenous metabolites produced by biochemical reactions under defined physiological conditions [9–11]. Li et al. analyzed the serum metabolome of diabetic patients via gas chromatography-mass spectrometry (GC–MS) and found that patients with pre-clinical, non-proliferative, and proliferative DR could be differentiated by metabolomics [12]. Some studies have also reported that regulation of the body’s metabolism can affect the development and outcome of eye diseases [13, 14]. Compared with plasma, ocular samples such as aqueous humor (AH) and vitreous humor are more relevant for studying the pathophysiology of retinal diseases. Nathan et al. suggested that pathological changes to vitreous metabolism in patients with DR underly disease features such as oxidative stress [15]. Young et al. collected vitreous samples from 42 patients and performed metabolomic analysis to distinguish between ocular inflammatory diseases [16]. They performed principal component analysis and were able to distinguish lens-induced uveitis from non-infectious uveitis with a sensitivity of 78% and specificity of 85%. The study showed for the first time that metabolomic analysis of vitreous humor samples could differentiate various types of vitreoretinal disease based on the different combination of metabolites present. In addition, AH has been used to detect metabolites of other eye diseases, including acute ocular hypertension, glaucoma, myopia, and AMD [17–21]. Aribindi et al. collected control and glaucomatous AH and found several unique cholesterol and glycosphingolipid species in a subset of patients with primary open-angle glaucoma or controls [20]. To the best of our knowledge, a metabolomic approach has not been applied to analyze samples of AH from patients with ME of different etiologies. In addition, the safety and convenience of AH collection compared with that of vitreous collection may increase the feasibility of its future clinical application. By generating a complete metabolomics profile of AH, we can increase our understanding of the potential role of molecular changes in the pathogenesis of ME.

This study aimed to investigate the metabolite profile of AH in patients with ME of different etiologies including AMD (AMD-ME), DR (DME), and branch retinal vein occlusion (BRVO) (BRVO-ME). We illustrate the underlying metabolic basis of AH for different etiologies across various ME populations and identify AH-derived metabolite biomarkers by liquid chromatography-mass spectrometry (LC/MS)-based metabolomics and a series of univariate and multivariate statistical analyses. We suggest that the “metabolic portrait” of different types of ME can improve the understanding of pathophysiological metabolic characteristics and various etiologies of ME based on the different profiles of metabolites present. Metabolic profiling of AH samples may help guide diagnosis and prognosis as well as determine treatment efficacy in various vitreoretinal diseases.

## Methods

### Study design

The study protocol was approved by the Ethics Committee of the Eye Hospital of Wenzhou Medical University (No. 2021-096-k-80-01). All patients in this study originated from the ophthalmology clinic and ward of the Eye Hospital. Patients were diagnosed with ME based on clinical manifestations combined with OCT, optical coherence tomography angiography (OCTA) and/or fluorescein fundus angiography (FFA) examination. Patients were excluded from the study if they had any history of intraocular surgery, trauma or systemic diseases other than hypertension. With the exception of patients with DME, no other subjects had any history of diabetes (Table 1). A sample of AH was collected from 60 patients with ME and 20 age- and sex-matched controls during routine intravitreal injection or anterior chamber puncture. After surface anesthesia, the eye was prepared aseptically according to routine procedures for ophthalmic surgery. The AH sample (approximately 0.1 mL) was manually aspirated into a 1.5 mL centrifuge tube. After collection, the sample was immediately stored at − 80 °C for later testing. Clinical information including age, sex, diagnosis, history of eye disease(s), medical history, preoperative visual acuity, and date of sampling was recorded for all patients.

**Table 1:** Patients’ clinical characteristics

Characteristic	AMD-ME	BRVO-ME	DME	CON
Eyes (cases)	20 (20)	20 (20)	20 (20)	20 (20)
Male/female	11/9	11/9	8/12	10/10
Age (years)	63.7	61.7	58.2	60.3
Hypertension	7 (35%)	8 (40%)	7 (35%)	9 (45%)
Diabetes	0 (0%)	0 (0%)	20 (100%)	0 (0%)
Cataract	80%	65%	75%	100%
Anti-VEGF (within 3 months)	0%	0%	0%	0%
Eye surgery (within 3 months)	0%	0%	0%	0%
Vitreous hemorrhage	0%	0%	0%	0%

*AMD* = age-related macular degeneration; *ME* = macular edema; *BRVO* = branch retinal vein occlusion; *DME* = diabetic macular edema; *CON* = control group; *VEGF* = vascular endothelial derived growth factor

### Metabolite extraction

Each AH sample (50 µL) was transferred to a centrifuge tube. After adding 200 μL of extract solution (acetonitrile:methanol = 1:1, internal standard mixture containing isotopic labelling), the sample was sonicated in an ice bath for 10 min and incubated at − 40 °C for 1 h, then centrifuged at 4 °C and 12,000 rpm (relative centrifugal force = 13,800×*g*, R = 8.6 cm) for 15 min. The supernatant was transferred to a new glass vial for analysis. A quality control (QC) sample was prepared by mixing equal volumes of supernatant from all samples.

### Liquid chromatography-tandem mass spectrometry (LC-MS/MS) analysis

LC-MS/MS analysis was performed by Biotree biotech Co., Ltd. (Shanghai, China) using high-performance liquid chromatography with a diode array detection system (Thermo Fisher Scientific). A UPLC BEH Amide column (2.1 × 100 mm, 1.7 μm) coupled to a Q Exactive HFX mass spectrometer (Thermo) was used for separation. The mobile phase consisted of 25 mmol/L ammonium acetate and 25 mmol/L aqueous ammonia hydroxide solution (pH 9.75) and acetonitrile. The working temperature of the automatic sampler was 4 °C, and the sample injection volume was 3 μL. A Q Exactive HFX mass spectrometer was used because of its ability to collect tandem mass spectrometry (MS/MS) spectra in information correlation acquisition mode under the control of the acquisition software (Xcalibur, Thermo). In this mode, the acquisition software continuously evaluates the full-scan MS spectrum. The electrospray ionization source conditions were as follows: sheath gas flow rate 30 Arb, Aux gas flow rate 25 Arb, capillary temperature 350 °C, full MS resolution 60,000, MS/MS resolution 7500, collision energy 10/30/60 in NCE mode, and spray voltage 3.6 kV (positive) or − 3.2 kV (negative), respectively.

### Data pre-processing and annotation

Raw data were converted to mzXML format using ProteoWizard and processed by an in-house program developed using R based on XCMS for peak detection, extraction, alignment, and integration. Then, an in-house MS2 database (BiotreeDB) was applied for metabolite annotation. The cut-off value for annotation was 0.3. Only metabolic features with a relative standard deviation less than 30% in QC samples were retained. Metabolic features detected in more than 50% of the samples were selected for further analysis, and missing values were recoded according to the 1/2 minimum. An internal standard-based normalization strategy was selected.

### Development of the diagnostic metabolic model

Differentially expressed metabolites (DEMs) were screened using univariate analysis (Mann-Whitney U test) and partial least squares-discriminant analysis (PLS-DA). Metabolites with variable importance in projection (VIP) ≥ 1, log_2_(fold change) > 1 and false discovery rate (FDR) < 0.05 were identified, where VIP was obtained from PLS-DA and FDR was obtained from the Mann-Whitney U test. Recursive feature elimination (RFE) was applied to these DEMs to identify optimal metabolites that could distinguish a specific disease from other diseases or healthy samples. The feature selection process was performed using the R package caret. The selected optimal metabolite combination was then used to train the gradient boosting machine to predict the likelihood of a new sample being a specific disease type or healthy (i.e., “BRVO-ME”, “AMD-ME” or “DME”). The gradient boosting machine was trained using the R package caret. The samples were stratified into the training cohort and test cohort at a ratio of 6:4.

### Statistical analysis

Principle component analysis and PLS-DA were performed to show the global metabolic differences between groups. Pathway enrichment analysis was performed on MetaboAnalyst (https://www.metaboanalyst.ca/) to obtain a significantly enriched Kyoto encyclopedia of genes and genome (KEGG) pathways. Venn diagrams were created using the R packages venn and UpSetR to identify common and specific features. Receiver operating characteristic (ROC) curves were created using the R package pROC to illustrate predictive power. All statistical analyses were performed in R version 3.6.0.

## Results

### Overview of data quality

**Fig. 1:**
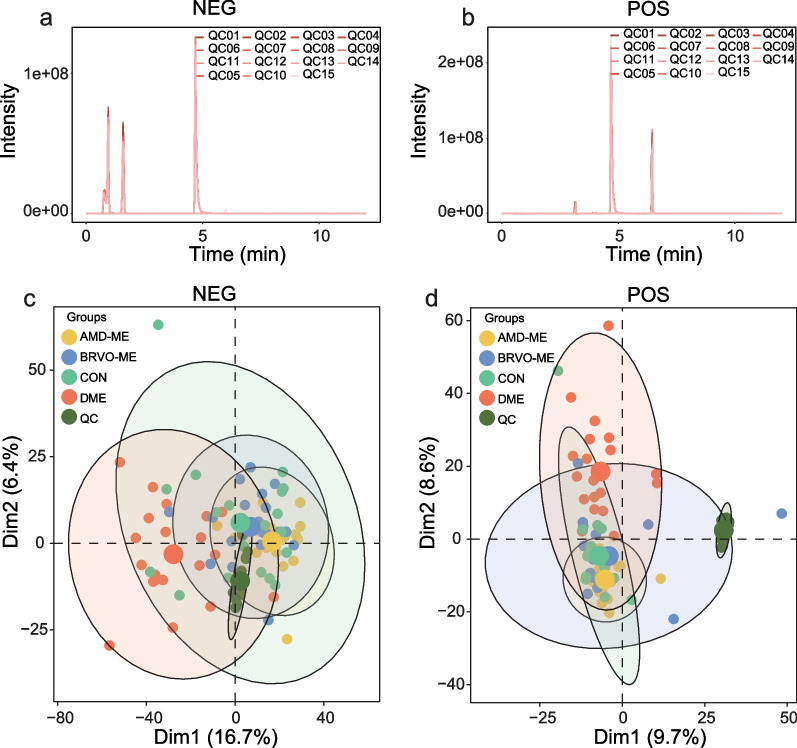
Quality control (QC) assessment of liquid chromatography-tandem mass spectrometry (LC-MS) analysis. Total ion chromatogram (TIC) of QC samples in negative ion mode (NEG, **a**) and positive ion mode (POS, **b**). Principle component analysis score plots for all aqueous humor samples in NEG (**c**) and POS (**d**) with different colored dots representing different groups of samples. AMD-ME, macular edema of age-related macular degeneration; BRVO-ME, macular edema of branch retinal vein occlusion; CON, control group; DME, diabetic retinopathy

To ensure data quality and reproducibility, the QC samples were applied to monitor the signal and instrumental deviation. As shown in Fig. 1a, b, the retention times and areas of the internal standard overlapped well in positive ion mode and negative ion mode for all QC samples. The characteristics detected in samples of the disease groups (AMD-ME, DME, and BRVO-ME) and the control group were subjected to principal component analysis (PCA), and obvious clustering of the QC samples was observed in both positive and negative ion modes (Fig. 1c, d). These findings demonstrated the stability of the detection processes, the reliability of data quality, and the stability of data acquisition.

### Differential metabolic profile of AH from patients with ME and healthy controls

**Fig. 2:**
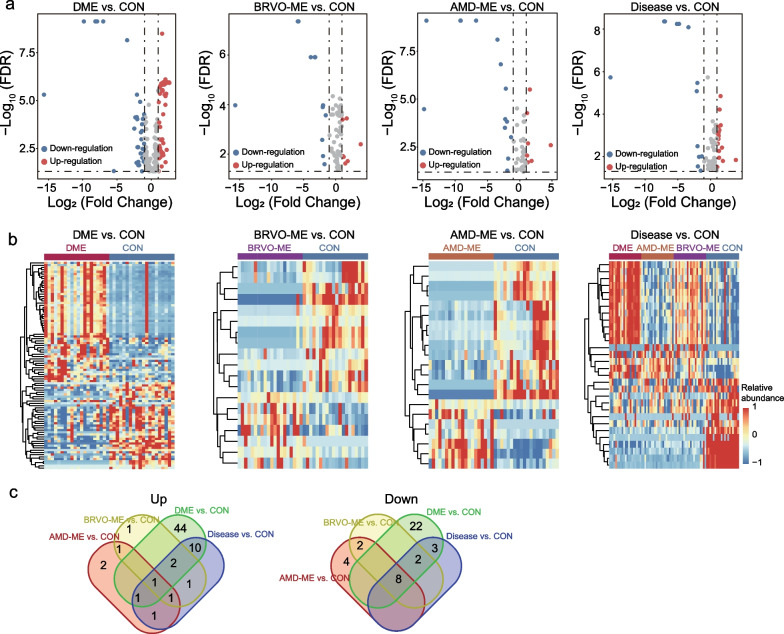
Identification of differentially expressed metabolites (DEMs) of aqueous humor from macular edema (ME) and control group (CON). Volcano plot (**a**) and heatmap (**b**) of differentially expressed metabolites for different disease groups and CON. **c** Venn diagram showing the overlap among DEMs between different disease groups and CON. AMD-ME, macular edema of age-related macular degeneration; BRVO-ME, macular edema of branch retinal vein occlusion; DME, diabetic retinopathy; FDR, false discovery rate

To investigate DEMs in the disease groups (AMD-ME, DME, and BRVO-ME) and the control group, we first performed multivariate analysis (PLS-DA) and identified metabolite features with VIP ≥ 1 as important variables for further analysis (109 metabolites in negative ion mode and 201 metabolites in positive ion mode). Next, we performed the Mann-Whitney U test to analyze these 310 metabolites and identified those with log_2_(fold change) > 1 and FDR < 0.05 as DEMs. Finally, a total of 93 metabolites (58 upregulated and 35 downregulated), 19 metabolites (seven upregulated and 12 downregulated), 21 metabolites (seven upregulated and 14 downregulated) and 30 metabolites (17 upregulated and 13 downregulated) were identified as DEMs in the DME, BRVO-ME, AMD-ME and ME groups, respectively, compared with the control group (Fig. 2a, b, Additional file 1: Tables S1–S4). Specifically, nine DEMs were shared by the three types of ME with different etiologies and controls, as well as all ME and control comparisons (one upregulated: clavulanic acid and eight downregulated: (+)-Setoclavine, Atropine, d-synephrine, (2*S*,4*R*,5*S*)-Muscarine, l-dopachromate, alpha-Methylphenylalanine, 2-(Formamido)-*N*1-(5-phospho-d-ribosyl) acetamidine, and 3,4-Dihydro-5-(5-methyl-2-furanyl)-2*H*-pyrrole). One DEM (one upregulated), 66 DEMs (44 upregulated and 22 downregulated), and six DEMs (two upregulated and four downregulated) were found to be BRVO-ME-specific, DME-specific and AMD-ME-specific, respectively (Fig. 2c).

### Metabolite pathway analysis

**Fig. 3:**
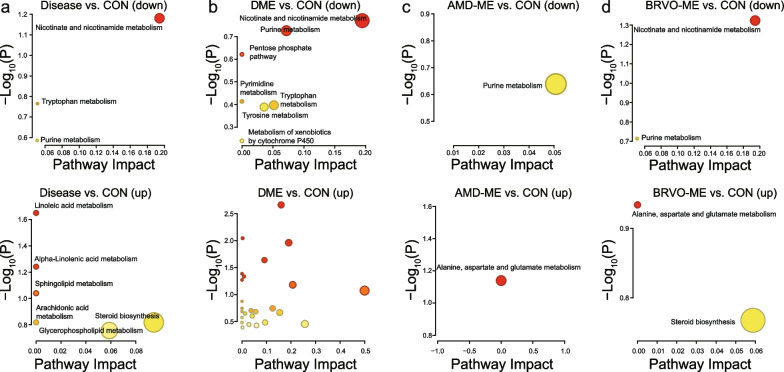
Functional characterization of differentially expressed metabolites (DEMs) of aqueous humor from ME (**a**), DME (**b**), AMD-ME (**c**), and BRVO-ME (**d**) compared with the CON group. *DME* diabetic macular edema, *AMD-ME* macular edema of age-related macular degeneration, *BRVO-ME* macular edema of branch retinal vein occlusion, *CON* control group, *ME* macular edema

The important metabolite pathways were subjected to pathway analysis using MetaboAnalyst 5.0; identified DEMs mostly belonged to lipid metabolism, mainly including nicotinate and nicotinamide metabolism, linoleic acid and linolenic acid metabolism, sphingolipid metabolism, arachidonic acid metabolism, and glycerophospholipid metabolism (Fig. 3, Additional file 2: Tables S5–S8). The identified DEMs of amino acid metabolism mainly included tryptophan, tyrosine, alanine, aspartate, and glutamate metabolism and, for a few DEMs, cytochrome P450, glucose, purine, and pyrimidine metabolism. Specifically, nicotinate and nicotinamide metabolism, tryptophan metabolism and purine metabolism were significantly downregulated in all ME patients (Fig. 3a). Linoleic acid and alpha-Linolenic acid metabolism, sphingolipid metabolism, arachidonic acid metabolism, glycerophospholipid metabolism, and steroid metabolism were significantly upregulated in all ME patients (Fig. 3a). Furthermore, amino acid metabolism, especially that of alanine, aspartate, and glutamate, was significantly upregulated in AMD-ME and BRVO-ME patients, while nicotinate and nicotinamide metabolism were downregulated in AMD-ME and BRVO-ME and ME groups (Fig. 3, Additional file 2: Tables S5–S8).

### Differential AH metabolites among ME of different etiologies

**Fig. 4:**
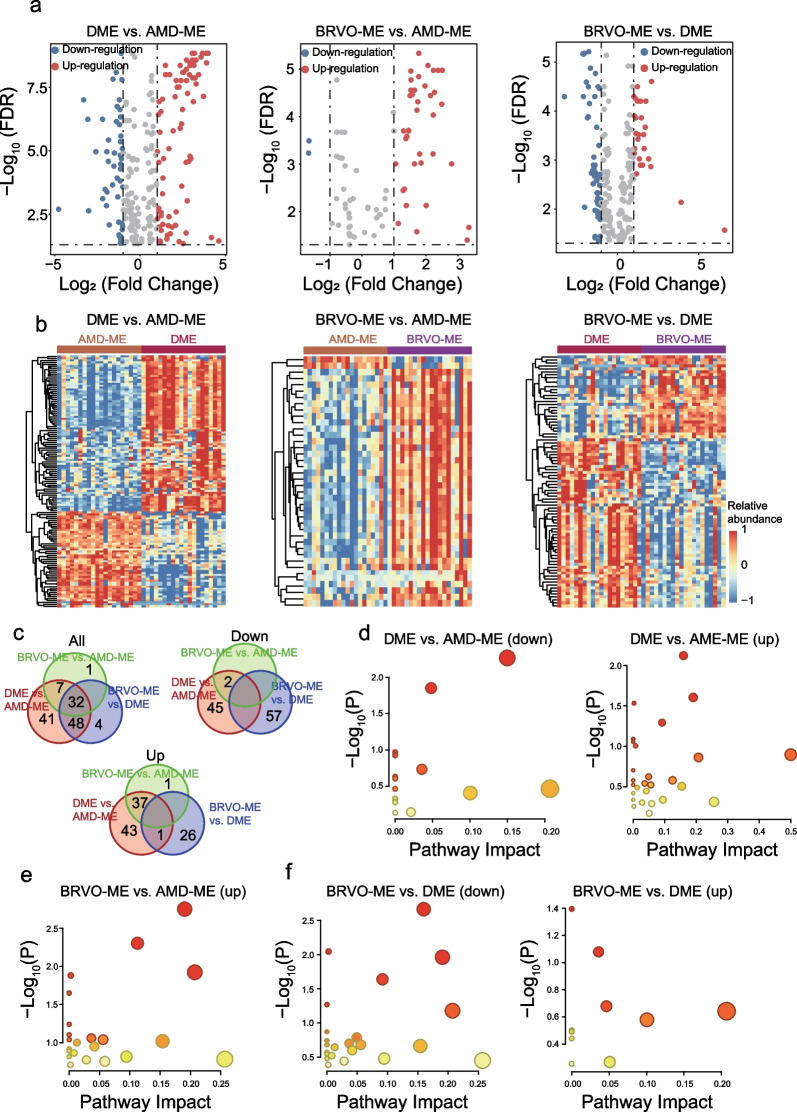
Identification and functional characterization of differentially expressed metabolites (DEMs) of aqueous humor among macular edema (ME) of different etiologies. Volcano plot (**a**) and heatmap (**b**) of differentially expressed metabolites for ME of different etiologies. Venn diagram visualizing the overlap among differentially expressed metabolites for ME of different etiologies (**c**). Functional characterization of DEMs of aqueous humor from DME and AMD-ME (**d**), BRVO-ME and AMD-ME (**e**), and BRVO-ME and DME (**f**). AMD-ME, macular edema of age-related macular degeneration; BRVO-ME, macular edema of branch retinal vein occlusion; CON, control group; DME, diabetic macular edema; FDR, false discovery rate

To further investigate the metabolic characteristics of AH in ME with different etiologies, the metabolic profiles of AH were compared, and overall differences in metabolites among the AMD-ME, DME, and BRVO-ME groups analyzed using PLS-DA. Characteristics with VIP ≥ 1 were taken as important variables (n = 312), and Mann-Whitney U test was performed to identify metabolite features with log_2_(fold change) > 1 and FDR < 0.05 as DEMs. Finally, a total of 40 metabolites (38 upregulated and two downregulated), 128 metabolites (81 upregulated and 47 downregulated) and 84 metabolites (27 upregulated and 57 downregulated) were identified as DEMs in BRVO-ME compared with AMD-ME, DME compared with AMD-ME and BRVO-ME compared with DME, respectively (Fig. 4a, b, Additional file 3: Tables S9–S11). Specifically, 32 DEMs were shared by the three types of ME (Fig. 4c). Further analyses showed significant differences in the metabolic pathways of AH derived from AMD-ME, DME and BRVO-ME and identified etiology-specific altered metabolites, including 39 for AMD-ME and 79 for DME (Fig. 4d–f, Additional file 4: Table S12). The enrichment results showed that carbohydrate metabolism including the citric acid cycle, glyoxylate and dicarboxylate metabolism, pyruvate metabolism and glycolysis/gluconeogenesis was upregulated in BRVO-ME patients compared with DME and AMD-ME patients (Fig. 4d–f, Additional file 4: Table S13). Remarkably, all 23 different metabolic pathways were upregulated in BRVO-ME compared with AMD-ME (Fig. 4d, Additional file 4: Table S14; Additional file 5: Fig. S1).

### Development and evaluation of a diagnostic metabolic model

**Fig. 5:**
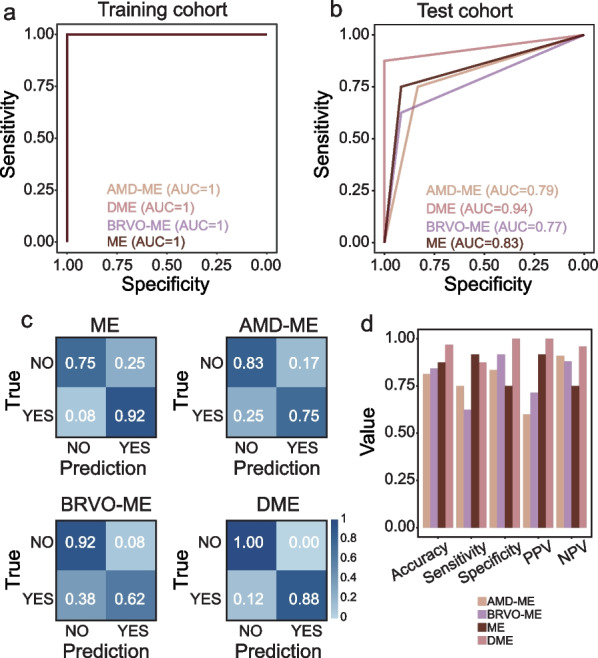
Performance evaluation of the diagnostic metabolic model. Receiver operating characteristic (ROC) curves of the diagnostic metabolic model in the training cohort (**a**) and test cohort (**b**). **c** Confusion matrices showing predicted outcomes generated by the diagnostic metabolic model in the test cohort. **d** Performance measurements of the diagnostic metabolic model illustrated by the five indices. AMD-ME, macular edema of age-related macular degeneration; BRVO-ME, macular edema of branch retinal vein occlusion; DME, diabetic macular edema; ME, macular edema; NPV, negative predictive value; PPV, positive predictive value

To search for optimal metabolite biomarker(s) for diagnostic prediction from DEMs, an RFE variable selection procedure was applied to the discovery dataset; 60 metabolites were identified as an optimal combination. The diagnostic model effectively distinguished ME from healthy controls with an AUC of 1 (Fig. 5a). For each etiology of ME, the diagnostic model had an AUC of 1 in the discovery dataset (Fig. 5a). When tested in the test cohort, the diagnostic model distinguished ME from normal samples with an AUC of 0.83 (Fig. 5b). For different disease groups, the diagnostic model had an AUC of 0.79 for AMD-ME detection, 0.94 for DME, and 0.77 for BRVO-ME (Fig. 5b). To test the discriminatory power of the model, we applied the diagnostic model to each disease group (Fig. 5c). The model consistently achieved comparable predictive performance as measured by accuracy, sensitivity, specificity, positive predictive value (PPV) and negative predictive value (NPV) (Fig. 5d). These results highlighted the robustness and stability of the AH-derived metabolic model for detection and differentiation of ME.

## Discussion

Diet, blood glucose, serum cholesterol, and triglyceride level are risk factors for fundus diseases, showing the importance of studying the metabolomes of these molecules to identify molecular biomarkers related to these risks [19, 22]. Retinal diseases are increasingly associated with metabolic dysfunction, so the study of systemic and local metabolic biomarkers may offer opportunities for early detection of disease and tracking disease progression [23]. In this study, the associations between AH metabolites and disease have been highlighted, including DEMs for each disease group (AMD-ME, DME, and BRVO-ME) and the control group and between the three etiologies of ME. Moreover, the possible pathways responsible for DEMs have been analyzed in depth, a disease prediction model using DEMs established, and the potential value of the model discussed.

The AH fills the anterior chamber of the eye and serves important functions in a normal intraocular homeostatic environment. Analysis of AH may provide insight into the pathophysiology of retinal diseases. Metabolites of eye diseases have been detected in AH samples in previous studies, and disorders in phospholipids, cholesterols, sphingolipids, and ceramides have been found in acute/chronic glaucoma studies [20, 24, 25]. AH has also been used to identify myopia-related metabolic changes. There are 40 metabolites that may be related to myopia, and 20 of them have shown significant differences at various stages of myopia [26]. In this study, 93, 19, and 21 DEMs were found in the DME, BRVO-ME and AMD-ME groups, respectively, compared with the control group. A total of 40 metabolites, 128 metabolites and 84 metabolites were identified as DEMs in BRVO-ME compared with AMD-ME, DME compared with AMD-ME and BRVO-ME compared with DME, respectively.

Despite the similar retinal structural changes in ME of different etiologies shown on OCT, the internal molecular-biological mechanisms were different, illustrating that despite similar retinal structural changes in ME of different etiologies shown on OCT, the internal molecular-biological mechanisms varies. In addition, nine novel common DEMs in three ME groups were identified that have not been detected previously (one upregulated DEM: clavulanic acid and eight downregulated DEMs: (+)-Setoclavine, Atropine, d-synephrine, (2*S*,4*R*,5*S*)-Muscarine, l-dopachromate, alpha-Methylphenylalanine, 2-(Formamido)-*N*1-(5-phospho-d-ribosyl). These metabolites may merit further investigation as key molecules in common ME pathogenesis. Among them, clavulanic acid, a naturally occurring powerful inhibitor of bacterial beta-lactamases, may cause generalized exanthema impetigo with severe laryngeal edema [27]. It would be very interesting to see whether there is a previously unreported causal relationship between clavulanic acid and EM. To fully understand biologically meaningful patterns of these identified DEMs in ME, we carried out metabolic pathway analysis to explore pathway-based metabolomics features.

Here, we found that the identified DEMs of ME mostly belong to lipid metabolism (mainly nicotinate and nicotinamide metabolism, linoleic acid and linolenic acid metabolism, sphingolipid metabolism, arachidonic acid metabolism, and glycerophospholipid metabolism), followed by amino acid metabolism (mainly tryptophan, tyrosine, alanine, aspartate, and glutamate metabolism). Lipid metabolism, especially that of linoleic acid and linolenic acid, sphingolipid, glycerophospholipid, and steroid metabolism, were significantly upregulated in all ME patients (Fig. 3). Cholesterol is the precursor of many important steroids and was significantly upregulated in AMD-ME, DME, and BRVO-ME. Early AMD is characterized by rich deposits of extracellular cholesterol below the retinal pigment epithelium, called drusen, or in the subretinal space, called subretinal drusenoid deposits. These deposits may drive the progression of AMD [28]. In addition, the structure and function of the retina relies largely on fatty acid composition [29]. There is evidence from epidemiological studies and animal experiments that fatty acid composition of the retina is influenced by diet [30–32]. This would imply that modulation of lipid metabolism through diet and drugs may affect retinal EM, providing new strategies for intervention.

The occurrence and progression of ME has been associated with the VEGF family [6, 7]. Inhibition of VEGF is currently the best drug-based treatment for ME [8]. Unfortunately, clinical evidence shows that intravitreal anti-VEGF therapy is ineffective in some cases of macular edema. In this study, there were 40, 128, and 84 DEMs between BRVO-ME and AMD-ME, between DME and AMD-ME, and between BRVO-ME and DME, respectively. The differences between DEMs of three ME groups may explain some of the differences in the efficacy of anti-VEGF therapy. There is a need to develop more targeted drugs according to the pathophysiological characteristics of the disease. For example, studies have shown that improving systemic metabolism using drugs like pemafibrate protects retinal function in DR [33]. Several studies have shown that diabetes causes metabolic changes in tissues including the retina [34, 35]. Indeed, the identified metabolites were quite different between DME and AMD-ME/BRVO-ME. Surprisingly, the metabolic pathways were similar in the three ME groups (Additional file 5: Fig. S1). We speculate that macular edema causes similar pathologic pathway changes in the retina, but the specific metabolites vary with the primary cause of ME.

With DEMs and pathway analysis, we assessed the impact of multiple metabolites in ME. Using the DEMs as characteristic metabolites, we input the 60 metabolites with the highest precision to establish the prediction model for etiology of ME (Fig. 5). The ROC results showed that the model could predict DME with the highest accuracy followed by AMD-ME and BRVO-ME. One study revealed variations in pyruvic acid and pyroglutamic acid which are considered useful biomarkers for diagnosing diabetes [10], thereby positively supporting the application of our results. Metabolic analysis of AH may similarly be used for early diagnosis of ME etiology, and prediction of the efficacy of anti-VEGF treatment for ME.

## Conclusions

Our study performed metabolic analysis of AH in patients with ME of different etiologies. Our findings illustrate the underlying metabolic basis of AH from ME populations and determined that DEMs mostly belong to lipid metabolism, followed by amino acid metabolism. We revealed significant differences in the metabolic composition of AH from ME patients with AMD-ME, DME and BRVO-ME. A novel AH-derived machine learning-based metabolic model is proposed for the early detection of ME. These findings will help explore the molecular mechanism of ME and may provide new perspectives for early diagnosis and in the search for new therapeutic targets.

## Supplementary Information

**Additional file 1.** Differentially expressed metabolites in the macular edema (ME) compared with the control group. **Table S1–S4**. Differentially expressed metabolites in the DME, BRVO-ME, AMD-ME and ME groups compared with the CON group. AMD-ME, macular edema of age-related macular degeneration; BRVO-ME, macular edema of branch retinal vein occlusion; CON, control; DME, diabetic macular edema.

**Additional file 2.** Differentially expressed pathways in the macular edema (ME) compared with the control group. **Tables S5–S8.** Differentially expressed pathways in the DME, BRVO-ME, AMD-ME and ME groups compared with the CON group. AMD-ME, macular edema of age-related macular degeneration; BRVO-ME, macular edema of branch retinal vein occlusion; CON, control; DME, diabetic macular edema.

**Additional file 3.** Differentially expressed metabolites between macular edema (ME) groups. **Tables S9–S11.** Differentially expressed metabolites in BRVO-ME compared with AMD-ME, DME compared with AMD-ME and BRVO-ME compared with DME. AMD-ME, macular edema of age-related macular degeneration; BRVO-ME, macular edema of branch retinal vein occlusion; DME, diabetic macular edema.

**Additional file 4.** Differentially expressed pathways between macular edema (ME) groups. **Tables S12–S14.** Differentially expressed pathways in BRVO-ME compared with AMD-ME, DME compared with AMD-ME and BRVO-ME compared with DME. AMD-ME, macular edema of age-related macular degeneration; BRVO-ME, macular edema of branch retinal vein occlusion; DME, diabetic macular edema.

**Additional file 5: Figure S1.** Venn diagram showing the overlap among differentially expressed metabolic pathways among ME of different etiologies. AMD-ME, age-related macular degeneration; BRVO-ME, branch retinal vein occlusion; DME, diabetic macular edema; ME, macular edema.

## Data Availability

All data generated or analyzed during this study are included in this published article and its Additional files.

## Abbreviations

ME: Macular edema
AH: Aqueous humor
DR: Diabetic retinopathy
AMD: Age-related macular degeneration
OCT: Optical coherence tomography
OCTA: Optical coherence tomography angiography
FA: Fluorescein angiography
BRVO: Branch retinal vein occlusion
LC/MS: Liquid chromatography-mass spectrometry
DEMs: Differentially expressed metabolites
PLS-DA: Partial least squares-discriminant analysis
RFE: Recursive feature elimination
PCA: Principal component analysis
PPV: Positive predictive value
NPV: Negative predictive value
CON: Control group
