# Supplementary figures and images for: Metabolomic analysis of aqueous humor reveals potential metabolite biomarkers for differential detection of macular edema
Source: Eye Vis (Lond). 2023 Apr 1;10:14. doi: 10.1186/s40662-023-00331-8 (PMC10067239; doi:10.1186/s40662-023-00331-8)

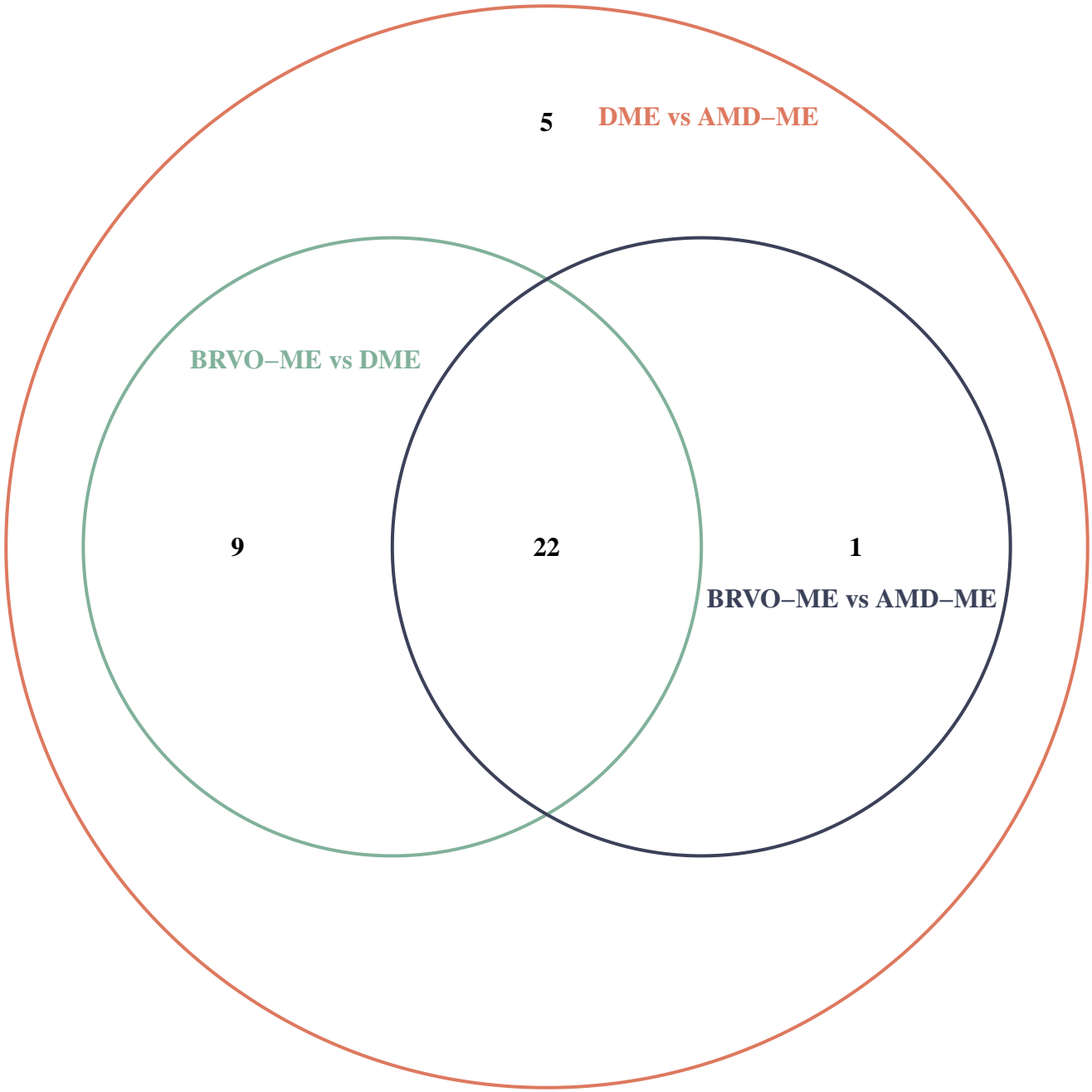

Supplement: Supplementary file 5 — Additional file 5: Figure S1. Venn diagram showing the overlap among differentially expressed metabolic pathways among ME of different etiologies. AMD-ME, age-related macular degeneration; BRVO-ME, branch retinal vein occlusion; DME, diabetic macular edema; ME, macular edema. [file 40662_2023_331_MOESM5_ESM.pdf]
